# Expansion and Function of Repeat Domain Proteins During Stress and Development in Plants

**DOI:** 10.3389/fpls.2015.01218

**Published:** 2016-01-11

**Authors:** Manisha Sharma, Girdhar K. Pandey

**Affiliations:** Stress Signal Transduction Lab, Department of Plant Molecular Biology, University of DelhiNew Delhi, India

**Keywords:** Armadillo, HEAT, WD40, TPR, PPR, ANK, tandem repeat, abiotic stress

## Abstract

The recurrent repeats having conserved stretches of amino acids exists across all domains of life. Subsequent repetition of single sequence motif and the number and length of the minimal repeating motifs are essential characteristics innate to these proteins. The proteins with tandem peptide repeats are essential for providing surface to mediate protein–protein interactions for fundamental biological functions. Plants are enriched in tandem repeat containing proteins typically distributed into various families. This has been assumed that the occurrence of multigene repeats families in plants enable them to cope up with adverse environmental conditions and allow them to rapidly acclimatize to these conditions. The evolution, structure, and function of repeat proteins have been studied in all kingdoms of life. The presence of repeat proteins is particularly profuse in multicellular organisms in comparison to prokaryotes. The precipitous expansion of repeat proteins in plants is presumed to be through internal tandem duplications. Several repeat protein gene families have been identified in plants. Such as Armadillo (ARM), Ankyrin (ANK), HEAT, Kelch-like repeats, Tetratricopeptide (TPR), Leucine rich repeats (LRR), WD40, and Pentatricopeptide repeats (PPR). The structure and functions of these repeat proteins have been extensively studied in plants suggesting a critical role of these repeating peptides in plant cell physiology, stress and development. In this review, we illustrate the structural, functional, and evolutionary prospects of prolific repeat proteins in plants.

## Background

A tandem repeat (TR) can be defined as the successive reiteration of a single repeat motif within a genomic sequence. The additional distinguishing features include the length and the number of repeating units as well as the sequence conservation among them.

A large fraction of eukaryotic genome is composed of internal repeats. Longer repeats comprising several repeat motifs in tandem are predominant in multicellular species. Subsequently, the presence of profuse repeating sequences depicts extra source of genome variability in eukaryotes compared to prokaryotes in order to offset slower generational period (Marcotte et al., 1999).

Evolutionarily, repeats are thought to originate by intragenic tandem duplication and genetic recombination (Andrade et al., 2001a,b). In addition to expansion, frequent duplications have caused variability in both the number and sequence of repeats even among orthologous genes. These internal TR are classified into three types based on the length of the repeats and their functions (Katti et al., 2000). Shorter repeats of nearly 2–20 amino acid residues are not proficient to exist as independent structural units and simply perform accessory function in repeat interactions. While, slightly longer repeats having nearly 20–40 amino acid residues prevailed as structurally independent units and some of them can efficiently fold into a three dimensional structure to mediate interactions among proteins. Furthermore, lengthier repeats comprising more than 100 amino acids are known to exist autonomously at both structural and functional level (Groves and Barford, 1999).

The aim of this review is to analyze the consensus, structure, and functions of nearly 40 residues long second type of TR proteins in plants. Repeat segments readily cluster into several multigene families within plant genome than in rest of the organisms (Marcotte et al., 1999). Various well-characterized plants repeat domain gene families include Armadillo (Groves and Barford, 1999; Coates, 2003), HEAT (Groves and Barford, 1999; Andrade et al., 2001a), Kelch (Adams et al., 2000), Tetratricopeptide repeats (TPR; Blatch and Lässle, 1999; Groves and Barford, 1999), Leucine rich repeats (LRR; Matsushima et al., 2005), Ankyrin (ANK; Batrukova et al., 2000), WD40 (Neer et al., 1994), and Pentatricopeptide repeats (PPR; Vetting et al., 2006). Taking advantage of their repetitive structure, repeat proteins have been shown to possess an intrinsic ability to bind peptides and thus act as an integral component of protein complexes. The TR often pack in a super-helical assembly that acts as a scaffold to mediate interactions among proteins (Conti et al., 1998). The fold and typical helical structure in these repeat ensembles regulate their affinity toward different ligands and partner proteins. Majority of repeat proteins adopt a distinctive structure with unique α or β helical strands exhibiting different groove area that provides them flexibility to bind diverse ligands and proteins. While repeat ensemble hold a well-defined structure, they are not significantly conserved at the sequence level. A small number of conserved residues within the primary sequence determine the precise folding of repeat domains (Bjorklund et al., 2006).

One of the coveted properties of TR proteins are that they almost always associate with additional functional domains. These accessory domains confer different biological roles to the proteins within the same gene family. This in part, explains the spectacular expansion of multigene TR families with characteristics motifs in plants in comparison to other species. The repeat proteins are assumed to facilitate transient interactions amidst proteins and associated functional domains allow them to play key role in diverse cellular processes conferring adaptation and tolerance to varying environmental conditions in plants. Numerous sequence-based studies have identified multigene repeat superfamilies’ in plants (Meyers et al., 1999; Andrade et al., 2001a; Nocker and Ludwig, 2003; Becerra et al., 2004; Mudgil et al., 2004; Rivals et al., 2006; Prasad et al., 2010; Sharma et al., 2014). Identification was followed by functional characterization of several members of these gene families during plant growth, development, and stress tolerance. In the following sections, we attempt to briefly explain the expansion, structure, and functions of different repeat gene families in plants.

## Expansion of Tandem Repeat Domain Gene Families in Plants

Higher plants, particularly *Arabidopsis* and rice genome encode for several gene families with domain repeats. Numerous whole genome analyses have effectively classified multigene repeat families in number of dicot and monocot species. Conducted studies have found nearly 108 and 152 genes representing Armadillo repeat family, in *Arabidopsis* and rice, respectively (Mudgil et al., 2004; Sharma et al., 2014). Similarly, 97 Kelch repeats were identified in *Arabidopsis* and 28 in rice by Sun et al. (2007). In addition, various databases have predicted more than 230 TPR containing domains in *Arabidopsis* and nearly 290 of these in the rice genome^1^. In a similar way, with 149 nucleotide-binding site (NBS-LRR) and 223 receptor-like kinases (LRR-RLKs), LRR repeats were identified as one of the largest gene family in *Arabidopsis* (Meyers et al., 1999; Gou et al., 2010). The number of ANK proteins has been found to be 175 in rice and 105 in the *Arabidopsis thaliana* (Becerra et al., 2004; Huang et al., 2009). Another large gene family with 237 representatives in *Arabidopsis* and closely 200 in *Oryza sativa* were found to be potential WD40 repeat gene family members (Nocker and Ludwig, 2003; Ouyang et al., 2012). Likewise, the PPR gene family also emerged as one of the largest gene family with nearly 466 genes in the *Arabidopsis* genome (Small and Peeters, 2000; Lurin et al., 2004), (**Table 1**).

**Table 1:** Putative number of repeats family proteins in various species. (Interproscan)

	Armadillo	HEAT	Ankyrin	PPR	TPR	LRR	WD40	Kelch
*Oryza sativa*	144	17	411	985	259	947	507	75
*Arabidopsis thaliana*	124	17	308	564	194	794	449	120
*Homo sapiens*	161	56	906	32	706	702	1216	195
*Danio rerio*	103	23	573	20	304	664	607	134
*Mus musculus*	127	41	708	18	342	539	833	142
*C. elegans*	16	11	161	11	57	74	195	16
*D. melanogaster*	48	5	300	11	147	363	439	33
*S. pombe*	7	3	14	4	26	9	121	1
*S. cerevisiae*	2	2	19	3	21	11	102	-
*Escherichia coli*	-	-	4	-	11	1	-	1
*Repeat length (aa)*	40–42	37–47	30–34	35	34	20–30	40	50
Type of Helices	α-helix	β-helix	helix-turn-helix	helix-turn-helix	helix-turn-helix	β strand-turn-α helix	β-propeller	β-propeller

From the above numbers, it is evident that the repeat domain families are much more abundant than the non-repeat gene families in plants. This abundance to a certain extent is attributed to internal duplications as well as to frequent genic duplication within gene families featuring TR (Ponting et al., 1999). In spite of this, ambiguity still persists on the degree of selection pressure, evolution impels upon TR gain or loss. The rapid proliferation of TR is specifically beneficial as it creates genetic variability allowing plants to adapt in adverse environmental conditions (Richard et al., 2008). It has been proposed that protein sequence conservation rather than frequent gain or loss of TR segment is an evolutionary preferred mechanism. Since, rapid change in sequence length and number disturb the overall structure of protein, and consequently its function (Schaper and Anisimova, 2015), the massive expansion of TR gene families in plants over the course of evolution suggests their prototypical role in plant growth and development. However, the mechanism underlying this expansion is not fully understood (Yang et al., 2010). Evolutionary events such as gene duplications, genetic recombination due to unequal crossing over, segmental duplications, and retrotransposition are known to play crucial role in the expansion and evolution of multigene families in higher plants (Fujii et al., 2011).

Apart from the above approaches to study gene family evolution, an alternative strategy to study gene duplication relies on the modular nature of proteins. Domains are the integral part of proteins that outline its structure, function, and evolution (Moore et al., 2008). The rate of evolution of the motifs within proteins is not as prompt as their rearrangement such as variation in number, combination, and pairing with a distinct domain across phyla. The domain reorganization and shuﬄing sporadically creates novel domains producing enormous diversity within proteins (Apic et al., 2001; Levitt, 2009; Yang et al., 2009; Moore and Bornberg-Bauer, 2012).

Genetic events such as domain gain and loss within large fractions of TR proteins in plants have lead to their emergence and subsequent diversification within the genome (Zmasek and Godzik, 2011). Emergence of repeated domains seems to be evolutionarily favored due to their involvement in plant-specific key functional processes such as transcription, protein binding, hormone, and secondary metabolite pathways.

Duplication events not only result into domain evolution but also increase plants complexity, necessitating equivalently intricate regulatory networks to sustain growth and development (Veron et al., 2007). In comparison to the established domains, relatively recent emerging repeat domains exhibit upsurge in intrinsic rearrangement that consequently enhance their protein binding affinity (Dyson and Wright, 2005). Owing to high binding affinity multitude of repeat motifs act as platforms for the assembly of protein complexes or facilitates transient interaction between proteins to regulate essential pathways.

Several reports have established the regulatory role of genes belonging to repeat families in different mechanisms such as signal transduction, cytoskeletal dynamics, cell division, apoptosis, light signaling, floral development, and many more transcriptional and signaling mechanisms.

## Structure and Detection of Repeat Families in Plants

Tandem duplications are an integral part of protein evolution. Multiple duplications and predominant presence of nearly 40–50 residues long fragments correspond to their regular secondary structure and proliferation particularly in eukaryotes (Kippert and Gerloff, 2009). While there is keen interest in identifying the biological functions of TR proteins prior to that we also need to investigate their tertiary structure and phylogenetic origin. The structural biology of various repeats proteins has already been deduced using X-ray crystallography and NMR studies.

**FIGURE 1:**
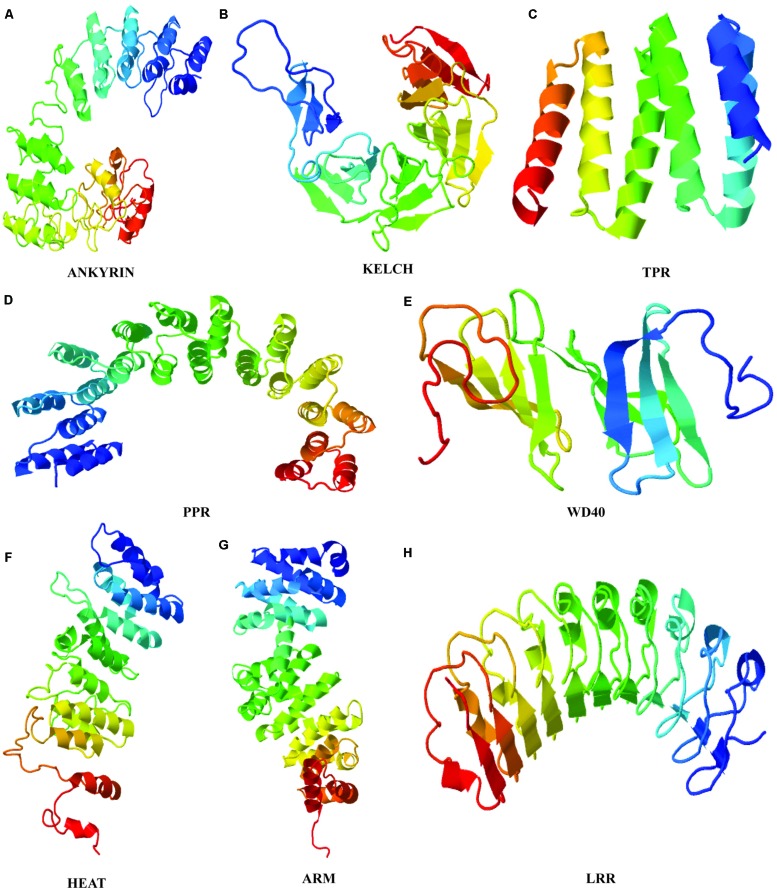
**Tertiary structures of plant repeat proteins. (A)** Ankyrin repeat domain of BDA1 (AT5G54610), (Yang et al., 2012). **(B)** Kelch repeat domain of BSU1 (AT1G03445) **(C)** TPR repeat domain of AT1G01320 **(D)** PPR domain of PPR1 (AT1G06580) **(E)** WD40 domain of ATG18 (AT1G03380) **(F)** HEAT domain of ILITHIYA (AT1G64790) **(G)** ARM domain of Arabidillo-1 (AT2G44900) **(H)** LRR domain of RLK7 (Receptor-like-Kinase 7). The amino acid sequence of only the repeat domain was used to generate the model from HHpred (http://toolkit.tuebingen.mpg.de/hhpred) using default parameters.

Identification of repeat units based on sequence analysis is a strenuous task that can be simplified with the use of structural approach because even in highly diverged sequences, the structure and functions are often preserved (**Figure 1**). TR confer several structural and functional advantages on proteins, that reflect in their variability. The repetitive motifs are the structural units within proteins that fold into different secondary structure arrangements such as linear arrays or as superhelical rod like arrangements (Andrade et al., 2001b). This three dimensional solenoid like structure provides an enlarged surface to facilitate binding with diverse molecules (Andrade et al., 2001b). The structural arrangement implications on functions of many proteins repeat families have been extensively studied. Structural elucidation of proteins of a repeat family provides sufficient evidence for the evolutionary mechanisms of their proliferation. The repeat families can be classified based on the number of assembly forming repeats and their structure. The characteristic open and closed structural configuration displayed by repeat proteins are created due to different length of the repeat motifs present in these proteins. The most studied type of TR proteins in plants are >∼42 amino acid long Armadillo repeat family.

Armadillo repeat domain is composed of one short and two relatively longer α-helices. The two longer helices are packed against each other with the shorter helix of ∼8 residues placed perpendicular to them to form a continuous superhelix or solenoid like conformation (Andrade et al., 2001a). Whereas, closely related HEAT repeats comprises two anti-parallel α-helices (**Figure 1**). Phylogenetic studies have predicted a common evolutionary origin for both Armadillo and HEAT repeat families. In animals, together they actively participate in nuclear protein transport complex (Malik et al., 1997; Cingolani et al., 1999). Structurally, HEAT and Armadillo possess similar superimposable grooves and helices to carry out protein interactions (Andrade et al., 2001a). Besides, both Armadillo and HEAT repeat families’ share same set of conserved residues that shape their hydrophobic cores (Andrade et al., 2001a). Regardless of resemblances, the HEAT and Armadillo variants have few sequence and structural distinguishing features. For instance, the interaction-mediating groove formed by the last helix of Armadillo has a conserved Asparagine residue not observed in HEAT repeats (Conti et al., 1998; Andrade et al., 2001a). Considerable variability at the sequence level in both of these repeats family qualifies them to be a success at evolutionary level. Moreover, the structural constraints within the Armadillo (ARM) and HEAT repeat superhelices usually lies on the entire array and not on individual repeat which further allow them to attain flexibility to bind number of interacting partner proteins (Andrade et al., 2001a; Kippert and Gerloff, 2009). The evolutionary conservation and proliferation of both repeats in plants is perhaps due to this inherited plasticity to fulfill diverse interactions.

The α-helical multi repeat arrays have also been detected in 34 amino acids long TPRs (Sikorski et al., 1990). The ubiquitous occurrence of TPR repeats across eukaryotic and bacterial genomes indicate their rather ancient evolutionary origin and conservation than any other known repeat proteins (Ponting et al., 1999). Many repeats within TPRs are known to organize into a right-handed superhelix creating a large groove for ligand and molecule binding (Das et al., 1998). The 35 amino acids long PPR repeats family share substantial sequence similarity with TPR repeats resulting in resemblance at structural level as well (Ban et al., 2013; Yin et al., 2013). The crystal structure of PPRs shows them to exhibit a superhelical ribbon-like structural fold. This has been proposed that PPR motifs have emerged by the divergence of TPR motifs considering their relatively limited divergence and distribution across species. However, significant differences exist between the two repeat families in relation with the conserved residues present in each of the motifs (Small and Peeters, 2000).

Likewise, the crystal structure analysis of Kelch and WD40 repeat families have revealed that their structural motifs represent a four to seven stranded antiparallel β-sheet organized radially in a propeller arrangement (Ito et al., 1994; Sondek et al., 1996). The internal repeats within β-propeller assembly embody closed structures with interactions between N- and C-terminal repeats. In β-propellers, the site formed by the N-terminal repeats along the propeller axis acts as a ‘supersite’ for the preferential binding of various ligands and molecules (Russell et al., 1998).

It has been observed that some of the repeats ensembles attain a mixed structure constituting both α- and β-helices within them. The 33 amino acids long ANK repeats are the class of proteins representing a structure of two antiparallel α-helices followed by an antiparallel β-sheet (Gorina and Pavletich, 1996). The L-shaped structure formed by close packing of the two different helices is further stabilized by hydrogen bonds between residues of adjoining repeats. Moreover, the side chains of the inner α-helix together with left-handed bend of the stacking create an extended groove for binding polar molecules (Sedgwick and Smerdon, 1999). Similar to ANK, LRRs belongs to the class of repeats containing α- and β-helix in antiparallel manner resulting into a horseshoe shaped structure. In comparison to other repeat families, LRRs are slightly shorter, with unit length of ∼20 residues (Kobe and Deisenhofer, 1994). The association of LRR repeats between two helices results into a coiled coil having core made out of β-strands to its interior, giving rise to an enlarged protein-binding surface (Andrade et al., 2001b).

Commonly occurring repeat proteins across eukaryotic genome are enriched with imperfect TR. However, perfect and nearly perfect repeats also occur in significant number in the genome. Different mutations such as insertions, substitutions, deletions contribute to sequence imperfection within repeats. In contrast to imperfect repeats the 3D annotation of perfect repeats is challenging due to strong residual conservation at each position making it hard to distinguish between residues of internal and peripheral regions (Jorda et al., 2010). The degenerate nature of the protein sequence in TR cause ambiguity in the identification of homologous repeats. The repeat proteins usually contain multiple repeats and the domain prediction databases frequently mis-predict their boundaries and hence folds (Kippert and Gerloff, 2009; Sharma et al., 2014). Moreover, in cases where proteins are composed of related but different repeat motifs, domain servers fail to make a distinction and detect only typical repeats leaving many other undetected due to their high significance stringency threshold. Repeat detection sensitivity can be improved by using sequence alignments of the query sequences rather than individual sequence (Kippert and Gerloff, 2009).

Highly diverged repeats sequences even within the same family restrict their annotation through structural visualization. Several domain prediction databases exist for repeat detection using both sequence as well as structure. Repeats detection databases mostly rely on the iterative sequence search to derive HMM (Hidden Markov Model) query profile and relating it with a template HMM. However, many of these databases do not take into account highly degenerate repeats and many repeat units are left undetected. Nonetheless, new improved repeat prediction algorithms and databases have been introduced in the recent past to fill this gap. These databases employ different algorithms for *ab initio* repeat detection. Some finds periodicities within amino acid sequences and are useful to detect long TR without any insertions and deletions; a characteristic of fibrous proteins, e.g., REPPER (Gruber et al., 2005; Kajava, 2012). Another category of programs such as XSTREAM rely on short seed extension heuristics to effectively characterize TR of variable lengths having indels and substitutions (Newman and Cooper, 2007). T-REKS is also based on similar seed extension algorithm but in addition to that it employs K-means algorithm to effectively detect repeats of variable length. The utilization of both XSTREAM and T-REKS is not limited only for amino acid sequences but these can also be applied to the DNA sequences (Jorda and Kajava, 2009).

Some other databases such as RepeatsDB, provides a systemic annotation and classification of repeats based on their structure (Di Domenico et al., 2014). RepeatsDB is a collection of predicted repeat units derived from Protein Data Bank (Berman et al., 2013), which were subsequently curated using RAPHAEL (Walsh et al., 2012). Further, few dedicated databases have been developed for the identification of specific TR. LRR finder^2^ can predict LRR domain with its N- and C- terminus and several other additional features using high stringency parameters. Another widely used specific repeat prediction algorithm include HHpred and COACH for the prediction of individual HEAT and ARM repeats with enhanced sensitivity and low false positive rate (Kippert and Gerloff, 2009), (**Table 2**). Perhaps, these are the most improved databases among all as they utilize previously generated repeats alignment to derive sequence and HMM profile. The usage of these sequence profiles further enhances the certainty and selectivity of the identified repeat sequences (Kajava, 1998).

**Table 2:** Different databases available for repeat identification.

Name	Methodology	Specificity	Link
LRR finder	PSSM	LRR	http://www.lrrfinder.com/lrrfinder.php
RepeatsDB	Curated PDB dataset	All repeats	http://repeatsdb.bio.unipd.it/
RADAR	Trace matrix analysis	All repeats	www.ebi.ac.uk/Tools/pfa/radar/
HHpred	HMM-HMM comparison	All repeats	http://toolkit.tuebingen.mpg.de/hhpred
T-REKS	K-meanS based	All repeats	http://bioinfo.montp.cnrs.fr/?r=t-reks

However, irrespective of the progress in the computational approaches proposing efficient detection of repeats, only certain types of repeats are being covered. Future work involving specific studies of more repeat types from different genomes may lead to formation of a comprehensive resource as a means to understand biological meaning of the repeat sequences.

## Repeat Domain Families and their Versatile Functions in Plants

The importance of repeats in understanding biological functions can be defined not only by their multiplicity but also by their ability to confer binding with proteins and mediating structural interactions. The abnormally high conservation of TR with binding properties in plants suggests that any change in these TR (Tandem Repeats) structural units may abolish protein function, and therefore evolutionary less favored. Proteins containing repeated sequences have been implicated in a myriad of biological processes. Many of the essential roles reported include signal transduction events where LRR, WD40, and ARM repeats are intricately involved. LRR and PPR participate in the biological molecule modification. Among others, PPR, TPR, and WD40 are actively involved in the cellular biosynthesis. Apart from their role in primary metabolism a large number of protein repeats regulate plant secondary metabolism comprising abiotic and biotic stress stimuli (Schaper and Anisimova, 2015).

## Tandem Repeat Ensembles in Plant Development and Stress Response

### Armadillo Repeats

Repeat proteins have been extensively studied in animals as well as in plants and are associated with multiple regulatory processes. There are profound implications of TR mediated protein binding and coordination between these interactions during stress and hormonal signaling in plants. Some of the characterized plant repeats proteins have been associated with well-defined biological functions. A large number of ARM repeat proteins in plants often associate with an accessory functional domain (Mudgil et al., 2004; Sharma et al., 2014). The most frequent domain arrangement observed is the U-box/ARM (PUB/ARM) combination suggesting a role of large complement of ARM repeats proteins in protein sorting *via* ubiquitination. The protein degradation mechanism plays a crucial role in plants to achieve proteome plasticity to respond effectively under environmental stress conditions. Several proteins in the largely studied Armadillo repeat family has been functionally characterized. One of the foremost-characterized ARM proteins is *Brassica* ARC1 (ARM repeat-Containing 1). Self-incompatibility response in angiosperms averts self-fertilization and enables cross-pollination for the maintenance of genetic diversity. ARC1 is a U-box/ARM domain containing E3 ligase protein identified as a positive regulator of *Brassica* self-incompatibility (SI) response mediated by SRK/SLP11 (S-locus receptor kinase/S-locus Protein 11) components. Stigma expressing ARC1 was confirmed to be specifically interacting with the kinase domain of SRK through its seven C-terminal ARM repeats (Stone et al., 2003). The receptor kinase phosphorylates ARC1, which in turn gets activated and catalyzes proteasomal degradation of target proteins allowing rejection of self-incompatible pollen (Stone et al., 2003). Interestingly, few other unrelated U-box/ARM proteins were identified as the regulator of SI response in *Arabidopsis* suggesting functional redundancy among these proteins (Liu et al., 2007). The structural homolog of ARC1, AtPUB16 was identified to express in response to GA and salt stress in order to mediate self-pollination in *Arabidopsis* (Acosta et al., 2012). The above studies suggest a possible role of ARC1 related genes in hormonal and stress signaling pathways. Expression analysis shows a large number of PUB/ARM proteins are induced in response to abiotic stress conditions (Yee and Goring, 2009; Sharma et al., 2014). In a study to identify salinity tolerance genes in mangrove (*B. gymnorrhiza*), transcript levels of one of the PUB/ARM genes, BgBG55 was reported to be induced when subjected to salt stress (Banzai et al., 2002). In another study, dehydration regulatory PUB/ARM protein was identified in *Capsicum annum*, named as CaPUB1. Heterologus expression of CaPUB1 in *Arabidopsis* plants renders them to be sensitive toward drought and salinity (Cho et al., 2006). Moreover, the CaPUB1 orthologs in *Arabidopsis* AtPUB22 and 23 also show similar response toward salinity and drought stress treatments suggesting this class of ARM/E3 ligases function as regulators of multiple abiotic stress responses (Cho et al., 2006, 2008).

Phytohormone such as gibberellin regulates plant growth, seed germination, stem elongation, and fruit development (Swain and Olszewski, 1996). In potato, photoperiod responsive ARM protein (PHORI) was isolated as a constituent of gibberellin signaling pathway. Phenotype of PHORI antisense plants was observed to be identical to GA response deficient mutants. PHORI consists of nearly eight ARM repeats, essential for its differential localization from the cytosol to the nucleus in the presence of GA (Amador et al., 2001).

Plant hormone cytokinin promotes cell division, differentiation and are essential for plant growth and development. In *Nicotiana*, a receptor-like kinase, CHRK1 with chitinase-related sequence in the extracellular domain was identified as a regulator of endogenous cytokinin levels (Lee et al., 2003). To gain further insights of its functions, interaction analysis of CHRK1 through library screening identified NtPUB4 as its putative interactor protein. NtPUB4 is a U-box E3 ligase protein consisting seven ARM repeats. The interaction between the kinase domain of CHRK1 and NtPUB4 is essentially mediated by ARM repeats as previously observed in the ARC1/SRK complex in *Brassica* (Kim et al., 2003). This interaction between the receptor like kinase and E3 ubiquitin ligase/ARM protein strongly suggest their role in cytokinin homeostasis in plants.

Apart from ARC1, role of an additional PUB/ARM protein, the rice *SPL11* (*spotted leaf 11*) was established in plant disease resistance. SPL11 was isolated from an EMS mutagenized indica cultivar showing spontaneous plant cell death phenotype. In addition to that, *spl11* loss-of-function plants display-enhanced resistance to fungal and bacterial pathogens. These findings provided another level of evidence to suggest the role of PUB/ARM protein in the negative regulation of plant defense mechanism (Zeng et al., 2004). PUB13 was identified as nearly 70% identical to SPL11 and thus considered as its closest ortholog in *Arabidopsis*. As expected, their high conservation at the protein level also translates into similar biological functions. Analogous to SPL11, PUB13 also function as a negative regulator of plant cell death, ROS accumulation, flowering time, and defense against biotrophic pathogens in a salicylic acid dependent manner (Li et al., 2012).

Under nutrient deprivation conditions, plants modify their root architecture to explore heterogeneous soil regions for nutrients. Branching of lateral roots from primary roots in plants is one of the processes to efficiently acquire nutrients from the soil. Several intrinsic development regulators exist in plants that do not require any hormone and signal induction to initiate LR growth (Malamy, 2005). In *Arabidopsis*, two of the F-box/Armadillo repeats proteins; Arabidillo-1 and Arabidillo-2, function redundantly to promote lateral root growth. Evidences have shown that both *Arabidillo-1, -2* transgenic plants were not showing any distinct hypersensitivity or tolerance toward well-known root growth factors such as nutritional deficiency and auxin related hormonal signals suggesting that perhaps they modulate some “intrinsic” lateral root growth pathway (Coates et al., 2006).

To further uncover this mechanism, subsequent interaction studies of Arabidillo proteins have identified MYB93 protein as part of this complex (Gibbs et al., 2014). Role of an additional protein belonging to Armadillo repeat family, AtPUB9 was demonstrated in lateral root growth specifically under phosphate starvation conditions. The manifestation of lateral root phenotype by AtPUB9 is followed by its phosphorylation by a S-domain receptor kinase (ARK2). Further, it was observed that the lateral root growth defect phenotype observed in the *pub9/ark2* mutants was due to low auxin accumulation at the initiation sites (Deb et al., 2014).

Apart from their role in plant growth and development, several PUB/ARMs were also identified to mediate abiotic stress response in plants. Role of four *Arabidopsis* PUB/ARM proteins (PUB18, 19, 22, and 23) was established in ABA mediated drought stress response. The quadruple mutant conferred enhanced tolerance to water deficient conditions in comparison to each of the single and double mutant phenotype. However, *PUB18* and *PUB19* show an ABA-dependent response, *PUB22* and *PUB23* perform ABA-independent drought response (Seo et al., 2012). Considering the fact that a large number of PUB/ARM genes are present in plants; therefore, role of more ARM proteins is anticipated in abiotic stress and hormone signaling by undertaking ‘Omics’ based high throughput approaches.

### Ankyrin Repeats

The other class of commonly found repeat motifs in eukaryotes is the ankyrin repeat domain. As a characteristic of degenerate repeats only some of the amino acids in the ANK repeating units are conserved, although numerous hydrophobic positions are conserved that are necessary to sustain the secondary structure (Bork, 1993). The most abundant group of ANK proteins are known to consists of transmembrane domains (Becerra et al., 2004). Several ANK transmembrane proteins have been characterized in animals. In plants, one of the first characterized ANK proteins was AKR (*Arabidopsis* Ankyrin Repeat). The transcripts levels of AKR were found to be light dependent and it was associated with the regulation of chloroplast differentiation (Zhang et al., 1992). Subsequent study has identified the AKRP interacting partner protein EMB506. EMB506 also consists of five ANK repeats and the association of AKRP-EMB506 protein complex is essential for plant organogenesis and morphogenesis during developmental stages (Garcion et al., 2006). Another ANK protein essential for cell differentiation is TIP1 (TIP growth defective 1). TIP1 encodes for an S-acyl transferase, is required for the normal cell growth and root hair development in plants (Hemsley et al., 2005). An additional ANK and ubiquitin ligase gene XBAT32 regulates lateral root growth in an auxin dependent manner in *Arabidopsis* (Nodzon et al., 2004).

Function of another ANK protein, AKR2A was shown in the biogenesis of chloroplast outer envelope protein (OEM). AKR2A has a dual property of recognizing both the target OEM signal on the chloroplast OEM proteins as well as the receptor on the chloroplasts to mediate their sorting and targeting (Bae et al., 2008).

The BLADE-ON-PETIOLE1 (BOP1) encodes for a distinctive BTB/POZ with ankyrin repeats domain architecture. BOP1 plays a key role in plant morphogenesis by modulating meristematic activity in leaves by regulating class I *KNOX* gene expression (Ha et al., 2003, 2004). However, several studies have elucidated the function of ankyrin repeat proteins during plant growth and development stages. Few of them were also characterized to play regulatory role during stress conditions. The inward-rectifier K^+^ channel, AKT1 composed of several transmembrane domains and five ANK repeats toward its C-terminus. AKT1 plays a significant role in root K^+^ uptake (Hirsch et al., 1998; Spalding et al., 1999; Gierth et al., 2005; Rubio et al., 2008; Alemán et al., 2011). Later, role of this protein has also been suggested in transpiration and adaptation to water deprivation stress. The *akt1* mutants display ABA hypersensitivity and enhanced drought tolerance due to increased stomatal closure during water deficient conditions. Similar response was also observed in *AKT1* regulatory *cipk23* mutant plants suggesting that both proteins function as negative regulator of plant drought response (Cheong et al., 2007; Nieves-Cordones et al., 2012). An additional ankyrin repeat protein OXIDATIVE STRESS 2 (OXS2) has been implicated as an activator of stress response pathway. Functional disruption of OXS2 results in plants sensitivity toward oxidative stress conditions. Intriguingly, overexpression of OXS2 did not result in higher stress tolerance in plants, however; it resulted in early flowering in plants (Blanvillain et al., 2011).

Many of these proteins are also known to act as chaperones for *trans*-membrane proteins. Ankyrin repeat 2 (AKR2) was identified as an interactor of *Arabidopsis* 14-3-3 protein, GF-14λ as well as one of its interacting protein Ascorbate Peroxidase3 (APX3). This interaction network suggests a role of AKR2 in plant ROS scavenging and metabolism (Shen et al., 2010). Functional characterization of ankyrin repeats proteins across species have indicated a conserved role for them in protection against pathogen and disease resistance. Salicylic acid levels in plants are required to mount strong response against plant pathogens for disease resistance. The NPR1/NIM1 (Non-expressor of PR1/Non-inducible Immunity1) is an ankyrin repeat containing defense protein found in many plant species (Sedgwick and Smerdon, 1999). Loss of NPR1/NIM1 renders plants extremely susceptible to pathogen attack due to failure of transcription of PR-1 (pathogenesis-related) genes and onset of SAR (Systemic Acquired Resistance) within them (Cao et al., 1994, 1997). Special classes of *Arabidopsis* mutants that accumulate high levels of SA and consequently show increased resistance toward diseases have been identified. ACD or Accelerated cell death is one of such mutants, which show increased SA accumulation and localized cell death under biotic stress (Rate et al., 1999; Brodersen et al., 2002). However, not all of these classes of mutants are directly induced by SA; rather their expression is somehow related to SA signaling. Conversely, expression of ACD6, a putative ankyrin repeat and transmembrane region containing protein was reported to be directly regulated by SA. The dominant gain-of-function mutant of *acd6-1* is influenced by SA and light to activate immune response involving spontaneous cell death to halt pathogen spread during infection. Plants with multiple copies of *ACD6* display better resistance toward *P. syringae*, indicating its role as a potential effector molecule of SA signaling pathway (Hua et al., 2003). Role of some of the rice ankyrin repeat protein was also established in the plant immune response. The ankyrin repeats domain containing XA21 binding protein 3 (XB3) is a class of E3 ubiquitin ligase, which forms a protein complex with receptor-like kinase (XA21). The XB3 is essential for sustaining steady state level of XA21 inside the cell to confer resistance against bacterial blight causing *Xanthomonas oryzae* in rice (Wang et al., 2006). Similarly, another ankyrin repeat-RLP (Receptor-like proteins) complex was recognized to mediate plant immune response. The SNC2 (suppressor of NPR1, Constitutive 2) is a receptor-like kinase complex that interacts with an ankyrin repeat domain containing transmembrane protein BDA1 (*bian da*) to activate downstream defense signaling components (Yang et al., 2012). An additional ankyrin domain containing rice protein OsPIANK1 was suggested to positively regulate rice basal defense against *Magnaporthe oryzae* attack (Mou et al., 2013).

### Pentatricopeptide Repeat

Pentatricopeptide Repeat constitute one of the largest gene families in *Arabidopsis*. The elucidation of broader biological context of PPR gene family is progressively improving in plants. Many of these proteins are characterized for their role in plant growth and development. Such as the 14 PPR motif containing EMB175 perform indispensible role in the *Arabidopsis* embryo development. Mutation in *emb175* causes defects in cell division rate resulting in morphological arrest. EMB175 is predicted to be localized in chloroplast thus could potentially disrupt vital metabolic processes centered there to arrest earliest phases of plant development (Cushing et al., 2005). The 19 PPR repeat containing LOJ (Lateral Organ Junction) protein express specifically at the LOJs and meristematic regions suggesting its function in shoot apical meristem development. However, disruption of LOJ does not cause any morphologically distinct abnormality in plants indicating redundancy in its function (Prasad et al., 2005). Apart from development, majority of PPRs have been characterized as restorers of cytoplasmic male sterility (CMS) caused by aberrant mitochondrial genes that prevent pollen maturation. In petunia, the 14 PPRs containing *Rf* locus (Restorer of fertility) protein *Rf* –PPR592 is identified as a mitochondrial-targeted protein capable of restoring fertility to *rf/rf* CMS lines (Bentolila et al., 2002). Using microsynteny analysis, another fertility restorer gene (*Rfo)* from radish with multiple PPRs was cloned. *Rfo* (fertility restorer) was predicted to contain mitochondrial targeting signal peptide. Transformation of *Rfo* into Ogura (*ogu*) CMS in *B. napus* resulted into recovery of male fertile plants (Brown et al., 2003; Desloire et al., 2003). To further understand the molecular mechanism of CMS, restorer loci of the Kosena radish (*Rfk1* and *Rfk2*) were studied. The 16 PPR motifs containing ORF687 was able to restore fertility to *B. napus* Kosena CMS cybrid by modulating the expression of mitochondrial gene *orf125*, responsible for male sterility (Koizuka et al., 2003).

Many of the PPRs have been classified to perform organelle specific functions in chloroplast and mitochondria. In a screen to identify nuclear mutants with defects in the metabolism of plastid RNAs, two *hcf152* alleles were isolated. Chloroplast localized Hcf152 analogous to its maize ortholog CRP1, encodes 12 PPR motifs domain and is an RNA binding protein. The *hcf152-1* and *-2* are non-photosynthetic mutants having significant flaw in the expression of the polycistronic *psbB–psbT–psbH–petB–petD* transcription resulting in lack of production in the cytochrome b_6_f complex suggesting a potential role in processing of plastid RNA (Meierhoff et al., 2003). The maize PPR protein CRP1 was proven to influence gene expression by associating itself with specific mRNA molecule. Co-immunoprecipitation has confirmed the role of CRP1 as transcriptional activator of specific organeller mRNAs (Schmitz-Linneweber et al., 2005).

Molecular evidences have predicted the role of several PPRs in a subset of biotic and abiotic stresses. The *Arabidopsis* nuclear localized PPR protein SVR7 (SUPPRESSOR OF VARIEGATION 7) is essential for the translation of the chloroplast ATP synthase subunits. Further, *svr7* mutants were shown to accumulate higher levels of ROS and display increased sensitivity to H_2_O_2_ with decreased photosynthetic activity (Lv et al., 2014). Analysis of a mitochondrial PPR protein PGN (PENTATRICOPEPTIDE REPEAT PROTEIN FOR GERMINATION ON NaCl) was identified to positively regulate biotic and abiotic stress response. *Arabidopsis* plants with mutation in *PGN* display low resistance against necrotrophic fungal infections as well as toward ABA, glucose and high salinity (Laluk et al., 2011). Another mitochondrial PPR protein SLG1 (slow growth 1) display delayed shoot growth phenotype and negatively regulate ABA and drought stress response (Yuan and Liu, 2012). In a unique study the function of a mitochondrial PPR protein (PPR40) was established as a potential link between mitochondrial electron transport and regulation of stress and hormonal response. Loss of PPR40 results into enhanced accumulation of ROS, increase in lipid peroxidation and superoxide dismutase activity and disruption of several stress-responsive genes suggesting correlation between cellular respiration and stress adaptation in *Arabidopsis* (Zsigmond et al., 2008). ABA overly sensitive 5 (ABO5) encodes a pentatricopeptide repeat protein required for the splicing of *nad2* (NADH dehydrogenase subunit 2) intron 3 in mitochondria. Mutation in *abo5* results in lower transcripts level of stress-inducible genes, such as *RD29A*, *COR47*, and *ABF2* in plants. Additionally, *abo5* mutants also accumulated higher level of H_2_O_2_ in roots than in wild type and this was further enhanced on exposure to ABA (Liu et al., 2010). This suggests that possibly impaired mitochondrial function causing redox imbalance activates ABA signaling leading to severe growth retardation and abiotic stress sensitivity in plants. Subsequently, an additional mutant having disrupted ABA signaling and impaired mitochondrial function was identified as *ABA hypersensitive germination 11* (*ahg11)*. AHG11 is a PPR protein speculated to perform RNA editing on mitochondrial protein nad4. Similar to *ABO5, Ahg11* mutant plants also exhibit higher transcript levels of oxidative stress responsive genes. In a recent study, yet another mitochondrial RNA editing PPR protein named as SLO2, which was earlier identified to regulate plant growth has been linked with stress response (Zhu et al., 2012a,b, 2014). In comparison to the above discussed mitochondrial proteins, *slo2* mutants exhibit enhanced expression of stress responsive genes. Additionally, mutant plants display hypersensitivity to ABA and osmotic stresses during germination stages whereas, adult plants were shown to exhibit tolerance toward salt and drought stresses (Zhu et al., 2014). Thus, the above evidences supports that mitochondrial RNA editing events also play a vital role in plant stress responses (Murayama et al., 2012).

Although large sets of mitochondria/chloroplast PPR proteins are classified to perform diverse role in plant stress and development signaling, a subset of PPR proteins localized to other cellular compartments were also identified. A recent study has identified a nucleo-cytoplasmic localized PPR protein, SOAR1 (suppressor of the *ABAR*-overexpressor 1) as a positive regulator of drought, salt and cold stresses (Jiang et al., 2015).

### LRR Repeats

The plant receptor like kinases containing extracellular leucine-rich repeat motif (LRR-RLK) encompass the largest subfamily in plants. Moreover, this subfamily is also one of the most well studied signaling molecules in plants. Increasing number of studies has provided evidence for their role in myriad of plant development, hormone and stress response pathways. One of the earliest identified LRR-RLK is a typical plasma membrane associated BRI1 kinase (Brassinosteroid Insensitive 1). BRI1 consists of a signal peptide on its N-terminus and an extracellular domain consisting of 25 leucine-rich repeats involved in brassinosteroid perception (Li and Chory, 1997). Shoot apical meristem size is regulated by another LRR-RLK, CLAVATA1 (CLV1) by perceiving peptide hormone CLV3 (Deyoung and Clark, 2008).

The function of two LRR proteins LRX1 and LRX2 has been defined in cell wall formation. LRX are the leucine-rich repeat proteins with a C-terminal extension domain required for the anchoring of protein into the cell wall (Draeger et al., 2015).

A small class of LRR-RLK family comprising ERECTA (ER), ERL1 and ERL2 regulates stomatal cell differentiation in plants (Pillitteri and Torii, 2012). The ER proteins functions together with another LRR receptor-like protein TOO MANY MOUTHS (TMM) to regulate stomatal patterning in plant epidermis (Guseman et al., 2010). A number of LRR-RLKs are induced under biotic and abiotic stress conditions. One of the LRR-RLK (RPK1) was characterized to be multifunctional regulator of plant development and stress responses. Transcript levels of LRR-RLK, RPK1 (receptor like protein kinase 1) are increased under different abiotic stress conditions. Mutation in RPK1 results in ABA insensitivity and altered expression of various stress responsive genes in the plants suggesting a positive role of RPK1 in stress signaling (Osakabe et al., 2005).

Several studies have further elucidated the role of RPK1 in ROS homeostasis. Overexpression of RPK conferred drought and oxidative stress tolerance in *Arabidopsis* (Osakabe et al., 2010). In a later study, RPK1 was identified to regulate leaf senescence in ABA-dependent manner (Lee et al., 2011).

In a different analysis, one of LRR-RLK (GHR1) was functionally characterized as a regulator of the activation of S-type anion channels associated 1 (SLAC1) in guard cells. The activation of these anion channels is under the control of ABA and H_2_O_2_. The GUARD CELL HYDROGEN PEROXIDE RESISTANT1 kinases phosphorylate the SLOW ANION CHANNEL-ASSOCIATED1, which results in closing of stomata under water deficient conditions (Hua et al., 2012). In rice, OsGIRL1 (*Oryza sativa* gamma-ray induced LRR-RLK1) expression pattern was examined under different abiotic stress and phytohormones conditions. The transcripts levels of OsGIRL1 were significantly induced under salt, osmotic and heat stress conditions. Similar induction was observed on treatment with SA and ABA; however, upon JA treatment OsGIRL1 expression was significantly reduced. These responses suggests role of *OsGIRL1* as a putative receptor of stress signals in plants (Park et al., 2014).

Another class of highly studied LRR proteins is the NB-LRR (nucleotide binding) proteins. Plants have two-tier system to mount resistance against pathogen attack. Basal resistance is conferred by PAMP-triggered immunity (PTI), while a second line of defense; ETI (effector triggered immunity) requires R proteins to initiate hypersensitivity response (Cunnac et al., 2009). These R proteins display general domain architecture with a variable N-terminal domain, central NBS and several LRR repeats at their carboxy-terminal (Chisholm et al., 2006; DeYoung and Innes, 2006).

A diverse group of LRR proteins have been characterized to mediate abiotic stress responses. One of the LRR-RLK genes, Srlk was classified as receptor regulating salt stress response in roots of *Medicago truncatula*. Loss in Srlk function resulted in increased insensitivity of roots toward salt stress (deLorenzo et al., 2009). Functional characterization of LRR-RLK7 has confirmed it to regulate seed germination and oxidative stress responses in *Arabidopsis*. Loss of RLK7 causes slower germination whereas overexpression results into faster germination. In addition, *rlk7* mutants accumulate fewer amounts of ROS detoxifying enzymes (Pitorre et al., 2010).

Fewer stress regulatory LRR-RLK members have been characterized in monocots. One of the rice LRR kinase OsSIK1, transcript level was observed to be induced under multiple abiotic stress conditions. OsSIK1 specifically expresses in stem and spikelets in rice plants. RNAi lines of OsSIK1 are sensitive toward salt and drought stresses conversely; overexpression lines display higher tolerance toward these stresses (Ouyang et al., 2010). Similarly, OsSIK2 was also classified as exhibiting similar stress response. OsSIK2 expression was found to be upregulated under salt, drought, cold, dark, and ABA stress conditions. Transgenic plants overexpressing *OsSIK2* were observed to be more tolerant toward these stresses than the wild type plants (Chen et al., 2013). Another stress responsive LRR-RLK gene identified in rice is LP2 (leaf panicle 2). It was observed that various abiotic stress conditions inhibited expression levels of LP2 in rice. Overexpression of LP2 in transgenic plants leads to reduction in reactive oxygen species accumulation and higher sensitivity of plants to water deficient conditions. Further analysis determined the upstream and downstream elements of LP2 mediated stress-signaling pathways. C2H2 zinc finger transcription factor DST (DROUGHT AND SALT TOLERANCE) was identified as a regulator of LP2 transcription. Additionally, LP2 was also found to interact with three plasma membrane intrinsic proteins (OsPIP1;1, OsPIP1;3, and OsPIP2;3) widely known to play crucial roles in drought stress response. This study thus established that DST regulated LP2 regulate drought stress response possibly in an ABA-independent pathway (Wu et al., 2014).

### TPR proteins

Tetratricopeptide repeats are present across all species and are known to mediate protein interactions with partner proteins. A number of TPRs are known to be involved in plant stress and hormone signaling. Similar to PUB/ARM domain arrangement PUB/TPR is also reported to be involved in plant stress signaling events. In *Arabidopsis*, ortholog of animal CHIP protein, AtCHIP is an E3 ligase consisting of three TPR repeats and a U-box domain. Variable temperature conditions cause the induction in AtCHIP expression. However, induction in AtCHIP expression caused higher sensitivity of plants toward temperature fluctuations. This was proposed that AtCHIP regulates some membrane channel proteins and remove damaged proteins under extreme temperature conditions, which apparently results into increase in electrolyte outflow in leaf cells (Yan et al., 2003). The function of an additional TPR gene NCA1 (no catalase activity 1–3) in abiotic stress conditions was established. NCA1 interacts with CATALASE2 (CAT2) through its C-terminal TPR repeats. Catalases are required for the maintenance of H_2_O_2_ homeostasis in plant cells. Sharp increase in catalase activity was observed in the presence of NCA1 that further requires binding of zinc ions to its N-terminal zinc finger domain. NCA1 is required for precise folding of catalase to maintain its functional state to withstand oxidative stress conditions (Li et al., 2015).

In *Arabidopsis*, ETO1 (ETHYLENE –OVERPRODUCER1) function as a negative regulator of ethylene biosynthesis by inhibiting the enzymatic activity of ACS5 (ACC synthase). ETO1 has a typical domain architecture consisting of a protein interacting BTB domain at the N-terminus and tandem TPR repeats on the C-terminus. The BTB domain is suggested to mediate interaction with E3 ligase AtCUL3 whereas; TPR domain binds with the AtACS5 protein to target this protein for proteasomal degradation (Yoshida et al., 2005). Function of an additional TPR protein is associated with ethylene signaling. The closest ortholog of tomato SlTPR1, in *Arabidopsis* named as AtTRP1 was characterized to influence plant development. *In vitro* and yeast two-hybrid assays have confirmed interaction between AtTRP1 with ethylene receptors LeETR1, ERS1, and NR. Overexpression of AtTRP1 also results in the altered ethylene signaling as well as change in expression pattern of certain auxin early responsive genes (Lin et al., 2009).

A different TPR repeat containing protein SPINDLY (SPY) displays overlapping role in gibberllin and cytokinin signaling pathway. SPY function as a negative regulator of GA signaling and loss of function mutants also exhibits insensitivity toward cytokinin suggesting an intermediary role of SPY in the two hormone signaling pathways. However, SPY is speculated to be involved in other regulatory pathways by mediating interactions with various other regulating proteins through its TPR repeats (Greenboim-Wainberg et al., 2005). Apart from phytohormone response, several TPR repeats are known to function in stress regulation. A novel plant specific TPR protein, TTL1 (TETRATRICOPEPTIDE-REPEAT THIOREDOXIN-LIKE 1) is being characterized to positively regulate ABA regulated stress responses. Loss of TTL1 function in plants resulted in sensitivity to salt and osmotic stress during seed germination and later developmental stages (Rosado et al., 2006).

In plant innate immune response, role of one of the TPR repeat protein known as SRFR1 was defined. SRFR1 (suppressor of rps4-RLD 1) functions negatively in resistance toward effector molecule AvrRps4. Loss-of-function analysis also confirmed that plants were insensitive toward (ETI) effector-triggered immunity elicitors (Kwon et al., 2009).

### WD40 Proteins

The large gene family of WD40 proteins are involved in a broad spectrum of crucial plant developmental processes. The regulation of anthocyanins and proanthocyanidins (Pas) biosynthesis is one of the most significant functions performed by WD40 repeat proteins. In *Arabidopsis*, a regulatory complex consisting of Transparent Testa2 (TT2) a Myb transcription factor, Transparent Testa8 (TT8) a basic-helix-loop helix domain, and Transparent Testa Glabrous1 (TTG1), a WD40 repeat protein regulate the flavonoid biosynthesis. Mutation in any of the Myb or HLH domain proteins in this complex results in the lack of pigmentation in flower and seed coat (Nesi et al., 2000, 2001; Baudry et al., 2004). However, loss of WD40 protein TTG1 results into many additional phenotypic defects in seed mucilage production and trichome and root hair formation (Koornneef, 1981; Walker et al., 1999).

Functional analysis of several other WD40 proteins suggested their involvement in chromatin based gene silencing and hence in gene regulation. CYCLOPHILIN71 (CYP71) is a WD40 repeat protein classified as a conserved histone modification regulator in chromatin-based gene silencing. The CYP71 gene regulates the activation of genes involved in meristem development. In the absence of CYP71, plants display growth defects such as meristem, reduced lateral organ development and reduction in root elongation. CYP71 directly interacts with histone H3 suggesting its role in chromatin based homeotic gene silencing (Li et al., 2007).

HOS15 is a WD40-repeat protein that mediates histone deacetylation of abiotic stress tolerance associated genes to repress their expression. Mutation in HOS15 also results in the development of cold hypersensitivity in plants. These results suggested a role of HOS15 in gene activation/repression by histone modification in plant acclimation to low temperature conditions (Zhu et al., 2008).

Study in wheat has identified the role of a WD40 repeat protein in abiotic stress conditions. TaWD40 containing tandem WD40 repeats is a stress inducible gene. Overexpression of TaWD40 in *Arabidopsis* resulted in enhanced tolerance to ABA, salt and osmotic stress in plants (Kong et al., 2015).

So far only a few WD40 proteins have been functionally characterized in plants. However, expression analysis and stress responsive *cis*-elements identification in many related studies suggest a potential role of WD40 proteins in stress regulation. For instance, the small subfamily of WD40 proteins SRWD (Salt Responsive WD40 repeats) was identified in rice. The tissue specific and stress specific expression profiling of these genes revealed them to be differentially expressed during salt stress (Huang et al., 2008). *SiWD40* (*Setaria italica* WD40) is a WD40 protein identified in foxtail millet. Promoter analysis of SiWD40 revealed the presence of two dehydration responsive elements that could be a putative binding site for a SiAP2 domain containing protein. These evidences indicate a role of SiWD40 in stress regulation driven by stress responsive *cis*-elements (Mishra et al., 2012).

## Conclusion and Perspectives

The rapid advances of sequencing technologies have allowed the analysis of various protein repeats across species. These analyses have drawn attention toward their typical conserved tertiary structure, evolution and particular expansion and multifunctionality in plants. The TR containing proteins are conserved not only in eukaryotes but also in bacterial and archaea species. The proliferation of TR usually occurs by repeat extension followed by internal duplication. However, the exact mechanism of the duplication pattern is unknown. Low conservation of TR units create difficulties in their identification. New bioinformatics tools and algorithms have been devised to ease this challenging task of repeat identification with high confidence. However, advanced tools need to be developed, which can be used to extract structural information of the repeat units whose tertiary structure is not defined. The prevalence of TR proteins in plants suggests their potential role in gene regulation in plant growth and development. One hypothesis suggest that the ability of TR proteins to bind to multiple ligands to establish interaction network in rapid response to external stimuli such as biotic and abiotic stress might play a role in their selection during evolution. Phylogeny based analysis have unraveled that the majority of repeat family proteins in plants are associated with different domain arrangements which could be correlated to the response to stress adaptation and tolerance. Future research is required to define the evolutionary trends in different species, the repeat motif features and their potential biological functions.

## Conflict of Interest Statement

The authors declare that the research was conducted in the absence of any commercial or financial relationships that could be construed as a potential conflict of interest. The reviewer Sebastian Pablo Rius and handling Editor Paula Casati declared their shared affiliation, and the handling Editor states that, nevertheless, the process met the standards of a fair and objective review.

## Footnotes

1 www.ebi.ac.uk/Tools/pfa/iprscan/

2 http://www.lrrfinder.com/lrrfinder.php
